# Large variability in the motility of spiroplasmas in media of different viscosities

**DOI:** 10.1038/s41598-018-35326-2

**Published:** 2018-11-20

**Authors:** J. F. Boudet, M. Mathelié-Guinlet, A. Vilquin, J. P. Douliez, L. Béven, H. Kellay

**Affiliations:** 1 U. Bordeaux, Laboratoire Ondes et Matière d’Aquitaine, UMR 5798 CNRS/U. Bordeaux, 33405 Talence, France; 2 UMR 1332, Biologie du Fruit et Pathologie, Univ. Bordeaux, INRA, 33882 Villenave d’Ornon, France

## Abstract

Spiroplasmas are bacteria that do not possess flagella and their motility is linked to kink propagation coupled to changes in the cell body helicity. While the motility of bacteria with flagellar motion has been studied extensively, less work has been devoted to the motility of spiroplasmas. We first show that the motility of such bacteria has large variability from individual to individual as well as large fluctuations in time. The Brownian motion of such bacteria both in orientation and translation is also highlighted. We propose a simple model to disentangle the different components of this motility by examining trajectories of single bacteria in different viscosity solvents. The mean velocity of the bacteria turns out to depend on the viscosity of the medium as it increases with viscosity. Further, the temporal fluctuations of the bacteria motility turn out to be very strong with a direct link to tumbling events particular to this bacteria.

## Introduction

Spiroplasmas are micro-organisms of the class Mollicutes which are considered as minimal bacteria. These Mollicutes have undergone drastic genomic reductions through evolution^1,2^. This gene loss is considered to be associated to their parasitic lifestyle and the remaining genes to represent the minimal set essential for their survival, multiplication, protection and pathogenicity in the host^3^. Spiroplasmas mostly infect arthropods (insects and crustaceans) and plants, and many species are pathogenic to their host. A better understanding of the virulence mechanisms of these pathogens is at present required in order to better counteract infections such as the citrus stubborn disease^4^, May disease^5^, tremor disease^6^, or human infections^7,8^.

Spiroplasmas are helical and motile^9–11^. While swimming in liquid media is associated with the presence of flagella in most bacteria^12,13^, spiroplasmas do not possess any flagellar structure and their motility includes bending, flexing, and change of helical sense propagating along the cell body (kink propagation)^14^. This type of locomotion appears to be well adapted for bacterial propagation in viscous media such as those colonized by spiroplasmas (lymph, phloem sap, joint fluid). In support of this assumption, the swimming speed of spiroplasmas was shown to increase with viscosity in the early 1980s^15^. The motility of these bacteria is actually linked to the generation of two temporally distinct ‘kinks’ travelling down the length of the bacterium^16,17^. The first kink triggers a change of spiroplasma helicity, e.g. a transformation of a right-handed into a left-handed helical structure. This change of helicity is associated with a change in orientation. The second kink restores the initial helicity and orientation. Upon the passage of the pair of kinks, the bacterium moves forward in the direction of its longitudinal axis^16^. Spiroplasma movements are thought to rely on structural changes of an internal contractible cytoskeleton^18–20^, and on the structural network formed by the bacterial homologs of actin MreB proteins^21^. The cellular and molecular organization of this internal cytoskeleton acting as a linear bacterial motor was resolved for both *Spiroplasma melliferum* and *S*. *citri*^19,20^.

Unravelling spiroplasma motility mechanisms may help in identifying potential drug targets to more specifically and more efficiently fight against these pathogens. However, to date, the relationship between motility and pathogenicity is unclear. Comparative studies of the pathogenicity of different *S*. *citri* strains, more or less affected in their motility, were hampered by the lack of quantification of the remaining motility capacities in these mutants^22^. More generally, studies to correlate motility and pathogenicity, or to understand the role of motility in spiroplasma biology, suffer from a lack of a simple method allowing the quantitative measurement of spiroplasma motility. It has to be mentioned that several studies reported the measurement of a mean swimming speed of spiroplasmas^14,15^. These studies bring important clues and significantly help understanding spiroplasma motility mechanisms and the dependence on the viscosity of the medium. Although the latter methods give important quantitative data, to our knowledge, no method described so far took into account the Brownian motion, which is necessarily critical for the displacement of bacteria with low Reynolds number. The variability in motility from bacteria to bacteria and versus time has not been addressed quantitatively either.

The present study aims at presenting a reliable method to accurately quantify spiroplasma motility in liquid media of different viscosities. *S*. *citri* was chosen as a model in this study as it is a pathogen of economic importance and can easily be cultivated *in vitro*. In addition, motility-deficient variants have been constructed or isolated for this species^23–26^. Our study and results should therefore be of interest to examine the potential correlation between motility and pathogenicity, and to identify drugs affecting spiroplasma fitness. A spiroplasma can roughly be represented schematically as a linear object with an internal motor ensuring a propagation speed along its axis and thus allowing a linear propagation of the bacterium. Nevertheless, the swimmer’s own movement in liquids competes with its Brownian diffusion^13,27^. On the basis of experiments conducted in media with different viscosities, we first study the effect of this parameter on spiroplasma paths. We demonstrate the change of average statistical properties of displacement and orientation of a spiroplasma population upon increase of the medium viscosity. A Langevin model of Brownian motion coupled to an internal motor is shown to be in agreement with experimental data. Our approach allowed us to analyze bacterial displacement, determine the mean speed of a single bacterium along with its variability from individual to individual as well as its temporal changes. Finally, a study of individual paths allows us to link the linear propagation velocity to the kink generation frequency, obtain the distributions of the velocity of the bacteria and identify the origin of the temporal fluctuations of their motility.

## Methods

*S*. *citri* GII-3 wild type has been isolated from the leafhopper vector *Circulifer haematoceps* captured in Morocco^28^. Bacteria are grown in SP4 medium^29^, in which fresh yeast extract was omitted, at a temperature of 32 °C. During the exponential growth phase of the bacteria, one volume of the culture is diluted with one volume of Methyl-Cellulose (MC) dissolved in SP4. Here, Methyl-Cellulose (from Sigma (M7027), Mol. Weight 14000 g/mol) was used to adjust the viscosity of the solutions. This was added in different proportions of 0, 0.25, 0.5, 0.75 and 1% by weight. The viscosities of the corresponding solutions were *η* = 1.2, 1.9, 2.9, 3.9 and 5.5 × 10^−3^ Pa.s. respectively. The viscosities were measured by using a capillary viscometer. Complementary measurements using the Brownian diffusion of micron sized particles (Polystyrene particles of 1.1 *μ*m in diameter from Sigma) to obtain their mean square displacement, their diffusion constant, and thus the viscosity of the solution using the Stokes Einstein relation were also carried out. These measurements turn out to be close to those using the capillary viscometer. Further, measurements using a cone-plate rheometer (Anton-paar) confirm these measurements and indicate that the viscosity of such solutions is roughly constant versus shear rate in the range 10 to 2000 s^−1^ suggesting that the solutions used are Newtonian. The elastic constants of these solutions are at least 5 orders of magnitude below their storage modulii again suggesting that the solutions are not viscoelastic.

Bacteria solutions were prepared between two sealed microscope slides, with a liquid thickness of 15 *μ*m, and observed under an Eclipse Ni (Nikon) microscope working in reflection and equipped with a dark-field condenser. The Nikon oil immersion microscope objective was a 60x with a N. A. of 0.80. Bacteria motion is recorded with a camera Nikon Digital Sight DS-Qi1Mc (1280 × 1024 pixels) at a frame rate of 10 frames per second (fps), leading to a time between frames ${\rm{\Delta }}t=0.1\,s$, with a spatial resolution of 0.106 *μ*m/pixel.

**Figure 1:**
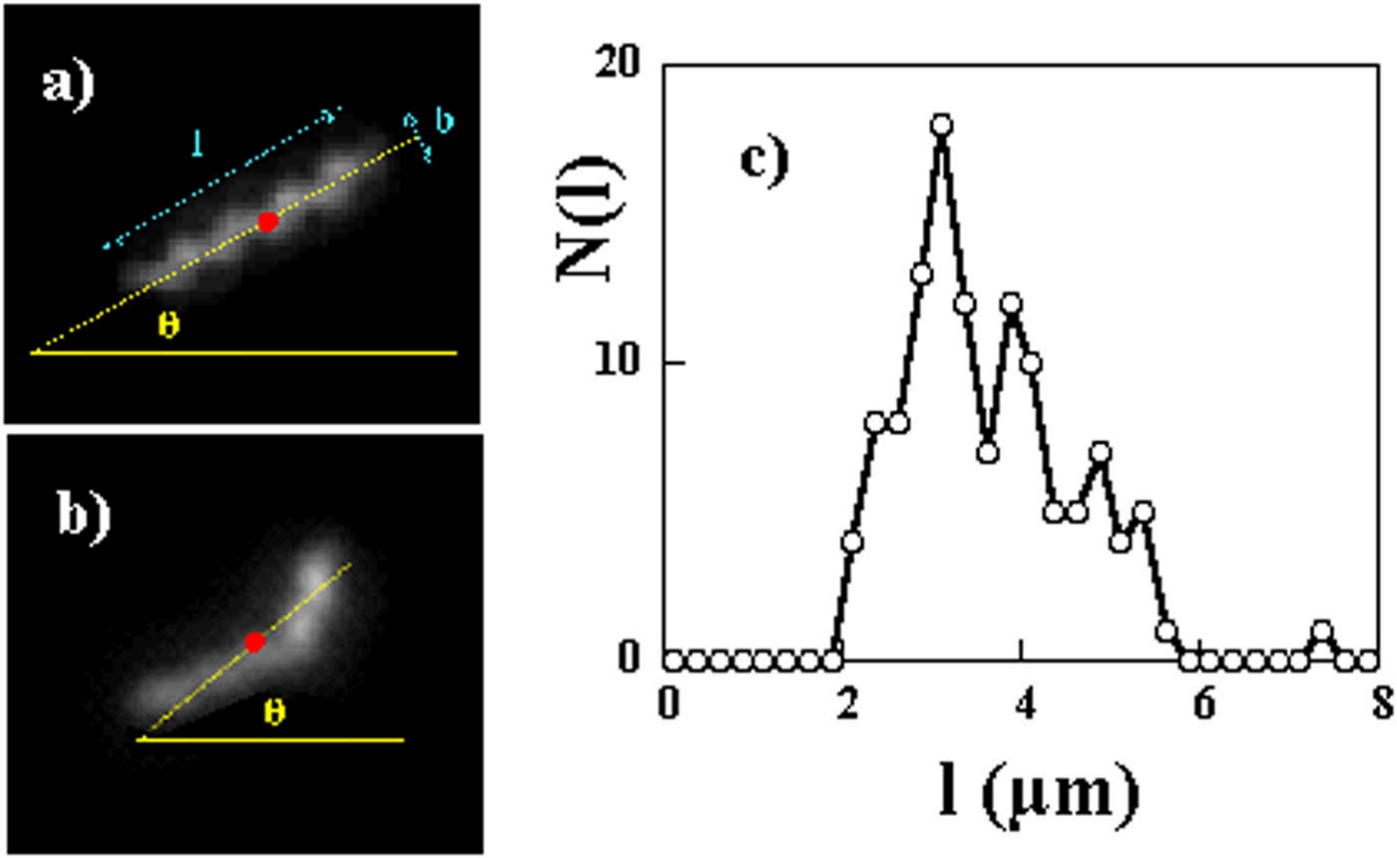
(**a**) Photo of a Spiroplasma along with the different parameters (length l, width b and major axis orientation *θ*). Note the apparent helicity. In this photo the length *l* = 2.9 *μ*m. (**b**) When the bacteria are not straight, their orientation is determined by the major axis of the intensity distribution. (**c**) Histogram of bacteria lengths.

From movies of the bacterial suspensions, the center of mass $\overrightarrow{r}(t)$ of a spiroplasma at the instant of time *t* was obtained as the center of mass of the scattered light intensity from the bacterium (Fig. 1a) using a home made Matlab code. The instantaneous velocity of the center of mass of the bacteria is obtained using finite differences of the displacement of its center of mass after a time ${\rm{\Delta }}$*t*:

$$\overrightarrow{V}(t)=\frac{\overrightarrow{r}(t+{\rm{\Delta }}t)-\overrightarrow{r}(t)}{{\rm{\Delta }}t}$$

The orientation *θ*(*t*) of the bacterium is obtained from the orientation of the long axis of the scattered light intensity. In all cases, in absence and in presence of a kink, as in Fig. 1a,b, the distribution of the light intensity is approximated by an ellipse and the angle of the major axis of the ellipse is taken as its orientation.

From microscopy images, the length of the bacterium *l* and its width *b* can be estimated. The value of *b* was found to be roughly *b* = 0.6 *μ*m. The length of the bacteria varies between 2 and 6 *μ*m; Fig. 1c shows the distribution of these lengths.

## Measuring the Velocities of Bacteria

This section examines the variation of the motility of the bacteria versus solution viscosity as well as its variability from bacterium to bacterium. In fact, the motility of the considered bacteria may have large temporal fluctuations. It is well known that bacteria such as *Escherichia coli* can run and tumble leading to large fluctuations in their instantaneous velocity^12^. Little is known about such an effect in *Spiroplasma* swimming. We will come back to this point below.

**Figure 2:**
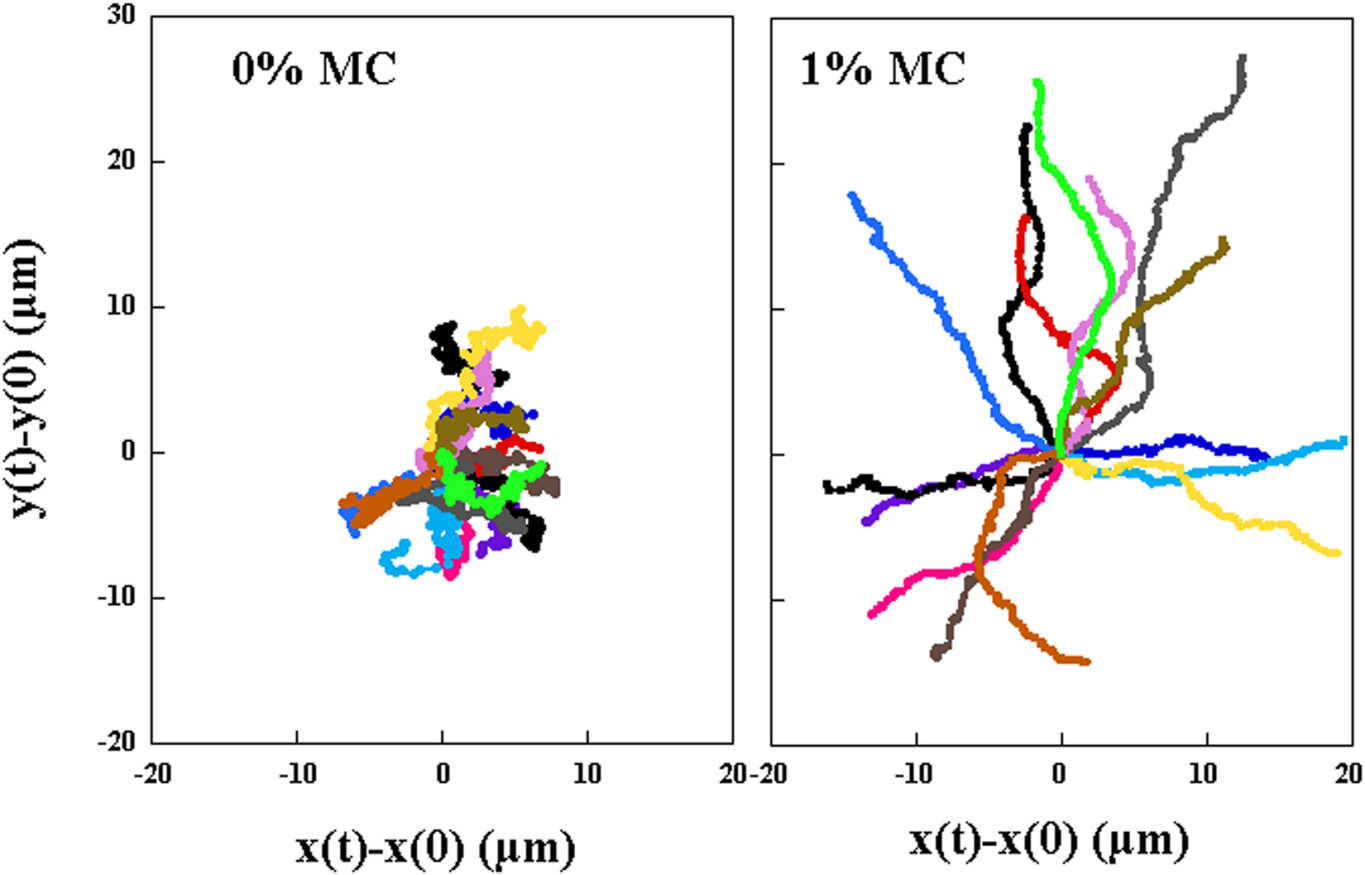
15 examples of center of mass trajectories of duration 10 *s* for two different concentrations of MC. The origin of the trajectories was set to (0, 0).

The influence of the viscosity on the motility can be appreciated in Fig. 2 where several trajectories are represented. The initial position is arbitrarily fixed at the origin of this graph. For the low viscosity solutions, the trajectories show large fluctuations and short paths while the more viscous solutions allow for smoother trajectories with a clear persistent motion. It is clear from this illustration that Brownian motion dominates the movement of the center of mass of the bacteria for low viscosity solutions while the persistent motion due to the motility of the bacteria dominates over the Brownian noise for the higher viscosity solutions.

**Figure 3:**
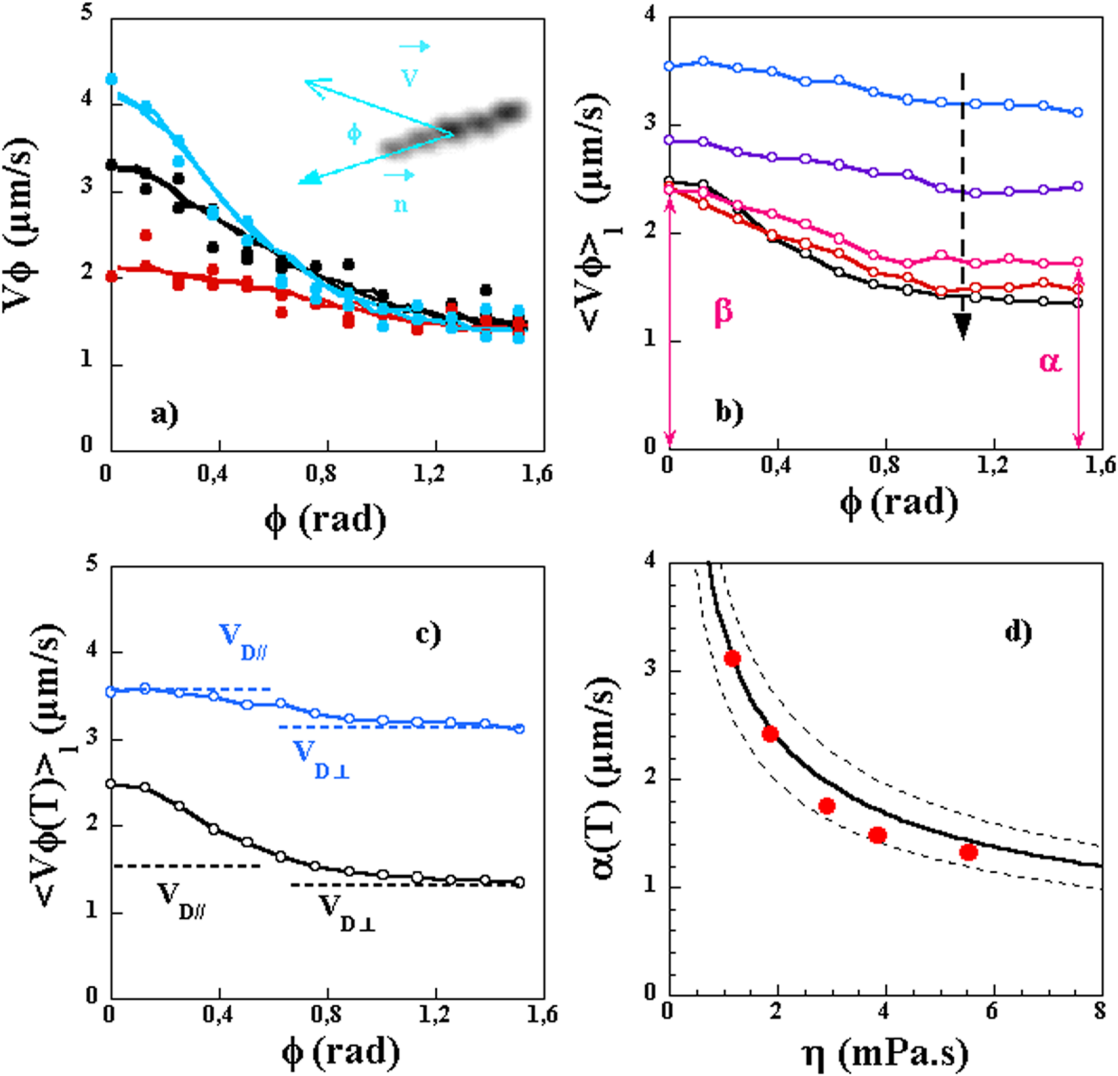
(**a**) ${V}_{\varphi }$ versus $\varphi$ for 3 different bacteria of comparable lengths in a solution of 1% MC. While for $\varphi =0$ the velocities are different, for $\varphi =\pi /2$ the velocities are roughly similar. The inset shows an image of a bacterium along with its velocity vector and its orientation. (**b**) Average velocity (over time and different lengths) ${\langle {V}_{\varphi }\rangle }_{l}$ for different concentrations of MC: 0, 0.25, 0.5, 0.75 and 1% MC from top to bottom. (**c**) ${V}_{\varphi }$ for a low viscosity (blue) and a high viscosity solution. For the low viscosity solution, the small angle value is dominated by the Brownian motion and allows to obtain the longitudinal diffusion constant. For the higher viscosity, both the swimming speed and the Brownian diffusion contribute to the value of ${V}_{\varphi }$ at small angles. For both solutions the value at high angles is given by the Brownian diffusion in the transverse direction. (**d**) Variation of *α* versus solution viscosity and comparison to expected value using a mean length of $l=3.5\,\mu {\rm{m}}$ (line). The dashed lines are the expected values for a length of 2 and 6 *μ*m.

In order to carry out a quantitative analysis of the motility of such bacteria, we first propose an analysis based on the direction dependence of the velocity of the bacteria. The velocity of the center of mass of the bacterium $\overrightarrow{V}$ is generally not collinear with the direction of the bacterium’s long axis $\overrightarrow{n}$: The bacterium does not move solely parallel to its orientation but has mobility in the transverse direction as shown in Fig. 3a. Let us call ${V}_{\varphi }$ the magnitude of the velocity vector $\overrightarrow{V}$ when it points at an angle $\varphi$ with respect to $\overrightarrow{n}$ (i.e. such that $\overrightarrow{V}\cdot \overrightarrow{n}=V\,\cos (\varphi )$) and averaged over time which we obtain as:

1 $${V}_{\varphi }=\sqrt{{\langle {V}^{2}(t)\rangle }_{\overrightarrow{V}\cdot \overrightarrow{n}=V\cos (\varphi )}}$$

The bacteria are followed over long periods of time and an average value over time of the square of their velocity in a direction defined by the angle $\varphi$ is obtained. Here, the brackets $\langle \mathrm{..}\rangle$ refer to a temporal average. At certain places the notation ${\langle \ldots \rangle }_{l}$ is used to indicate that the average is over time and different bacteria lengths. We use *V*^2^(*t*) because it is difficult to resolve the orientation of the bacteria, i.e. the direction of the vector $\overrightarrow{n}$ which is only known modulo 180°. Figure 3a shows ${V}_{\varphi }$ for three different bacteria with comparable lengths (*l* = 3.5 *μm*) in a solution of 1% MC. This velocity is maximum near $\varphi =0$ and decreases as $\varphi$ reaches 90°. Basically the bacteria have more mobility in the direction parallel to the long axis as compared to the perpendicular direction. This asymmetry is due to the presence of a motor velocity or swimming velocity primarily in the direction of the long axis. The origin of the mobility in the transverse direction is most probably Brownian translational motion which should be similar for the three bacteria shown as they have similar sizes. This is borne out by the measurements of Fig. 3a.

In order to analyze these effects in detail, we propose a simple model for the mobility of these bacteria. We assume that the bacteria have a motor velocity which has a well defined mean value and fluctuations around this mean value. Further, and because of their size, the bacteria are subject to Brownian motion. In principle, and if we note the motor velocity *V*_*m*_(*t*) of a single bacterium in the direction of the long axis $\overrightarrow{n}$, this velocity can be written as ${V}_{m}(t)=\langle {V}_{m}(t)\rangle +u(t)$, where $\langle {V}_{m}(t)\rangle$ is the time averaged motor velocity and *u*(*t*) is the fluctuating component with a mean of zero. The velocity $\overrightarrow{V}$ has another contribution coming from Brownian translational motion. The characteristic velocities of Brownian motion, over a short period of time ${\rm{\Delta }}$*t*, can be obtained as: ${V}_{D\perp }=\sqrt{4{D}_{\perp }/{\rm{\Delta }}t}$ and ${V}_{D//}=\sqrt{4{D}_{//}/{\rm{\Delta }}t}$. The two coefficients ${D}_{//}$ and ${D}_{\perp }$ stand for the translation diffusion coefficient in the parallel and orthogonal directions with respect to the long axis. Indeed, a rod like or ellipsoidal like particle has translational diffusion coefficients which are different in the two orthogonal directions. We model the bacteria as ellipsoids; other studies use cylinders for *E*. *coli* for example^30^ but ellipsoids turn out to be reasonable in our case. These coefficients for an ellipsoid in a medium of constant viscosity *η* read^31^:

2a $${D}_{//}=\frac{{k}_{B}T}{16\pi \eta b}\times \frac{(2{p}^{2}-1)S-2p}{{p}^{2}-1}$$

2b $${D}_{\perp }=\frac{{k}_{B}T}{32\pi \eta b}\times \frac{(2{p}^{2}-3)S+2p}{{p}^{2}-1},\,S=\frac{2}{\sqrt{{p}^{2}-1}}\times \,\mathrm{ln}\,(p+\sqrt{{p}^{2}-1})$$

Here *k*_*B*_ is the Boltzmann constant, $T=293^\circ K$ is the temperature (the microscopy experiments were carried out at 20 °C), and $p=l/b$ is the aspect ratio of the bacteria. Note that $\kappa ={D}_{//}/{D}_{\perp }$ is a measure of the anisotropy, is independent of the fluid viscosity, and increases with the aspect ratio *l*/*b*.

Let us assume that in the orthogonal direction the velocity is set only by the Brownian motion and in the longitudinal direction it is set both by the motor velocity and the Brownian motion, one can then write:

3 $${V}_{\varphi =\pi /2}={V}_{D\perp }=\alpha $$

4 $${V}_{\varphi =0}=\sqrt{{V}_{D//}^{2}+2\langle {V}_{m}^{2}(t)\rangle }=\sqrt{{V}_{D//}^{2}+2{\langle {V}_{m}(t)\rangle }^{2}+2\langle {u}^{2}(t)\rangle }=\beta $$

Here, we have assumed that the velocity of the bacteria is not correlated to its Brownian noise.

Figure 3a shows that the value of *α* is similar for the three considered bacteria which is as expected from our model. However, the value of *β* differs from bacterium to bacterium. Since the translational Brownian motion is similar for the three bacteria, their velocities must be different. What is therefore important in this figure is that the difference between the two directions is not the same for the three considered bacteria signaling an inherent variability of the motor velocity from one bacterium to the next.

Another important point is that such curves are very sensitive to the viscosity of the medium. In Fig. 3b, ${V}_{\varphi }$ is plotted for different solution viscosities and averaged over several bacteria and therefore different lengths as the velocity is independent of the length as we will see below. While for the higher viscosities the asymmetry due to the motor velocity is apparent, for the lower viscosity solutions it is much less obvious. The difference in velocity between the two orthogonal directions in the low viscosity solutions can actually be explained, at least in part, by the anisotropy of Brownian translational motion. Figure 3c shows that the value of *β*, for the lowest viscosity solution (0% MC) is given predominantly by the value of ${V}_{D//}$ while the value of *α* is given by the value of ${V}_{D\perp }$. This latter observation remains true for the higher viscosity solution but the value of *β* is much higher than ${V}_{D//}$ clearly indicating the contribution of the motor velocity. In fact, the low viscosity solution results can be used to estimate the diffusion coefficients in both directions of the bacteria and therefore obtain an estimate of the anisotropy coefficient *κ* which is independent of the viscosity.

From Fig. 3b, the values of *α* increase as the viscosity decreases as expected if the sole contribution is Brownian motion. This can be tested directly from the variation of *α* versus solution viscosity shown in Fig. 3d. This variation turns out to be in good agreement with the expected value from the diffusion constants. In an indirect manner, this measurement of *α* confirms that the bacteria are diffusing in a background with a viscosity similar to that of the solution as measured using a capillary viscometer or a rheometer excluding effects related to Non Newtonian aspects of the solution or possible entanglement.

The second observation is that the ratio *β*/*α* increases as the viscosity of the solution increases indicating an increase of the motor velocity. This ratio can actually be used to extract the motor velocity (more precisely ${\langle {V}_{m}^{2}(t)\rangle }^{\mathrm{1/2}}$) unambiguously and for each bacterium once the ratio $\kappa ={D}_{//}/{D}_{\perp }$ is known. This ratio is taken as that of an ellipsoid of aspect ratio *l*/*b* with the expressions for ${D}_{//}$ and ${D}_{\perp }$ given in equations 2a and 2b. This ratio is also estimated directly from the measurements of *β*/*α* for the lowest viscosity solution. All these estimates turn out to be in agreement with each other.

**Figure 4:**
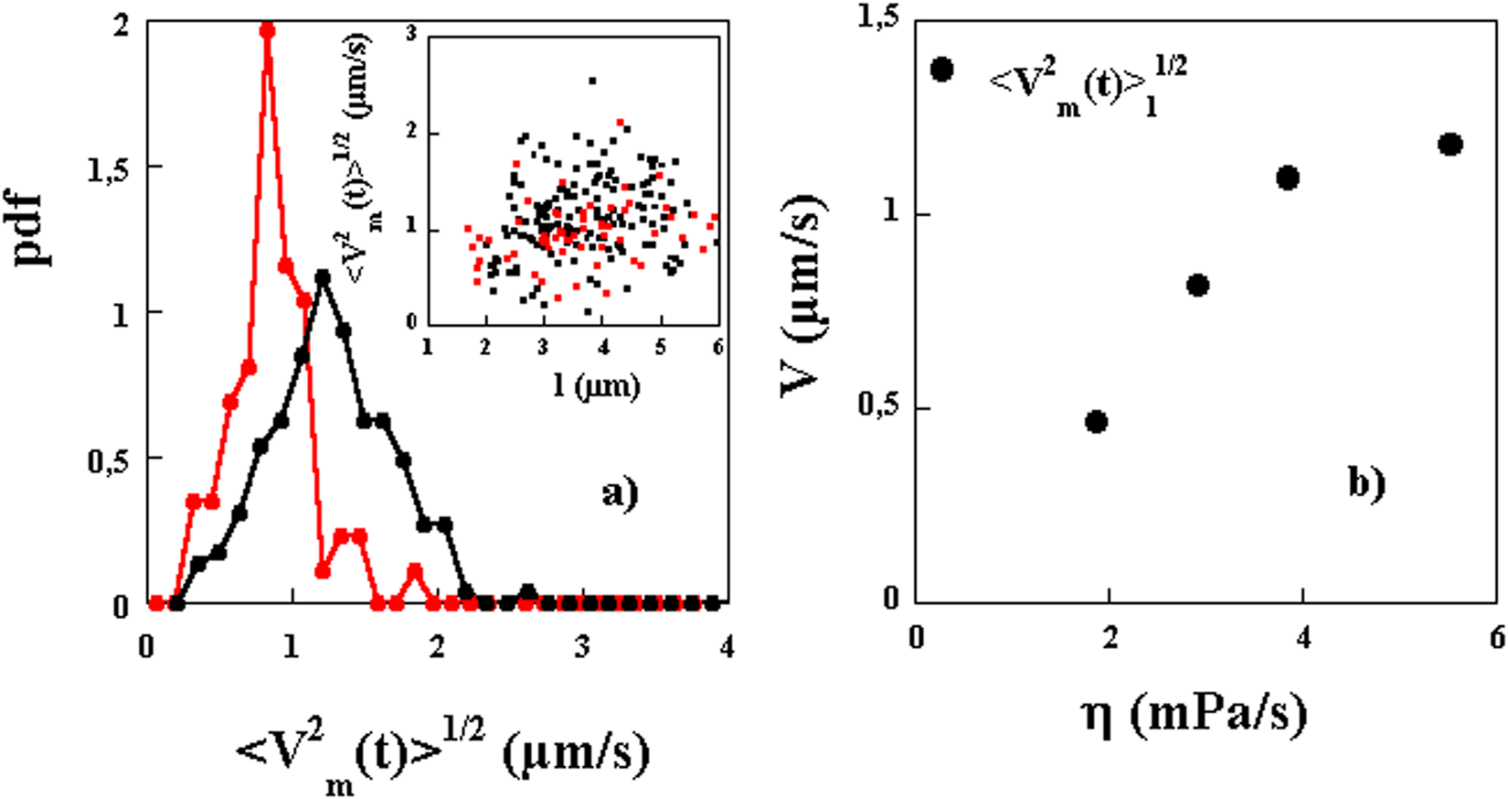
(**a**) Probability distribution functions of ${\langle {V}_{m}^{2}(t)\rangle }^{1/2}$ for two different concentrations of MC, 1% (black symbols) and 0.5% (red symbols). Inset: ${\langle {V}_{m}^{2}(t)\rangle }^{1/2}$ versus length of bacteria. (**b**) ${\langle {V}_{m}^{2}(t)\rangle }_{l}^{1/2}$ versus solution viscosity. The subscript *l* indicates that this velocity is averaged not only over time but over different lengths of the bacteria.

The values of the extracted motor velocities are shown in Fig. 4a for different lengths of the bacteria and two different viscosities. No significant variation with the length is observed but a large variability from one bacterium to the next (even for comparable lengths) is clearly seen. The Probability distributions of the measured velocities are also shown. While the distributions are broad, the mean value of ${\langle {V}_{m}^{2}(t)\rangle }^{\mathrm{1/2}}$ seems to be larger for the higher viscosity solution. The extracted values of ${\langle {V}_{m}^{2}(t)\rangle }^{\mathrm{1/2}}$, averaged over different lengths, are shown in Fig. 4b for different viscosities. These characteristic velocities increase as the viscosity increases. It should be stressed here that the extracted velocity ${\langle {V}_{m}^{2}(t)\rangle }^{\mathrm{1/2}}$ contains information about both the mean velocity $\langle {V}_{m}(t)\rangle$ as well as the fluctuations *u*(*t*). We will come back to estimating $\langle {V}_{m}(t)\rangle$ from a different analysis below so that the amplitude of the fluctuations can also be estimated.

To summarize these observations, the viscosity of the solution plays an important role in the persistent motion of the bacteria which is more important in the direction parallel to the bacteria’s long axis. Further, Brownian translational motion is present under all conditions and is strong enough to almost mask the motor velocity for the low viscosity solutions. The motor velocity of the bacterium seems independent of length and increases with the viscosity. This velocity has an inherent variability both from bacterium to bacterium and possible temporal variability.

While the above analysis allows to obtain the aggregate value of the mean velocity and the possible fluctuations around this mean value, we now propose a simple model of the motility of these bacteria. This model will allow us to obtain the mean velocities $\langle {V}_{m}(t)\rangle$ as well as give details about the diffusion constants of the bacteria.

## Statistical Analysis of the Trajectories of Individual Bacteria

The bacteria are modeled as rigid ellipsoids with a major axis of length *l*, a minor axis of length *b* and are assumed to possess rotational symmetry around the long axis. We assume that the bacteria are subjected to translational (center of mass) and rotational (of the long axis) Brownian motion along with a persistent mobility along the long axis with velocity *V*_*m*_(*t*). In the absence of inertia and assuming two dimensional motion, the equations of motion read:

5 $$\frac{d\overrightarrow{r}}{dt}=[{V}_{m}(t)+{\varepsilon }_{//}(t)]\cdot \overrightarrow{n}+{\varepsilon }_{\perp }(t)\cdot \overrightarrow{\tau }$$

6 $$\frac{d\theta }{dt}={\varepsilon }_{\theta }(t)$$

where $\overrightarrow{r}$ is the center of mass position, *θ* the orientation, $\overrightarrow{n}$ and $\overrightarrow{\tau }$ are respectively vectors parallel and orthogonal to the major axis of the bacteria and *t* is time. The terms ${\varepsilon }_{\perp }(t)$, ${\varepsilon }_{//}(t)$ and ${\varepsilon }_{\theta }(t)$ represent the effects of thermal noise which is assumed to be of zero mean and delta correlated in time. Their amplitudes are given by:

7 $$\begin{array}{l}\langle {\varepsilon }_{//}(t)\cdot {\varepsilon }_{//}(t+\tau )\rangle =2{D}_{//}\cdot \delta (\tau )\\ \langle {\varepsilon }_{\perp }(t)\cdot {\varepsilon }_{\perp }(t+\tau )\rangle =2{D}_{\perp }\cdot \delta (\tau )\\ \langle {\varepsilon }_{\theta }(t)\cdot {\varepsilon }_{\theta }(t+\tau )\rangle =2{D}_{\theta }\cdot \delta (\tau )\end{array}$$

where *δ*$(\tau )$ is the Delta function.

As indicated above, the brackets $\langle \ldots \rangle$ indicate an averaging over time. Recall that ${D}_{//}$ and ${D}_{\perp }$ stand for the translation diffusion coefficients in the parallel and orthogonal directions with respect to the long axis while *D*_*θ*_ stands for the angular diffusion coefficient and is given by^31^:

8 $${D}_{\theta }=\frac{3{k}_{B}T}{32\pi \eta {b}^{3}}\times \frac{(2{p}^{2}-1)S-2p}{{p}^{4}-1}$$

The equations of motion, eqs 5 and 6, allow to calculate the mean square displacement (MSD) and the mean square angular displacement (MSAD) in the case where the motor velocity ${V}_{m}(t)={V}_{m}$ is constant independent of time^13,27^, which amounts to neglecting the fluctuating component *u*(*t*):

9 $$MSD(t)=\langle {\overrightarrow{r}}^{2}\rangle =2({D}_{//}+{D}_{\perp })\cdot t+2\frac{{V}_{m}^{2}}{{D}_{\theta }}(t-\frac{(1-\exp (\,-\,{D}_{\theta }\cdot t))}{{D}_{\theta }})$$

10 $$MSAD(t)=\langle {\theta }^{2}\rangle =2{D}_{\theta }\cdot t$$

While the assumption of constant motor velocity is not justified, we will use it here to obtain estimates of the average value (over time) of this motor velocity for each individual bacteria. This will allow us to obtain the variability of the mean motor velocity from bacterium to bacterium and estimate the temporal fluctuations from a comparison with the velocities obtained in the analysis presented in the previous section. Note that the effects of the presence of temporal fluctuations, i.e. *u*(*t*), if delta correlated in time, will only act as an additional noise similarly to ${\varepsilon }_{//}(t)$.

**Figure 5:**
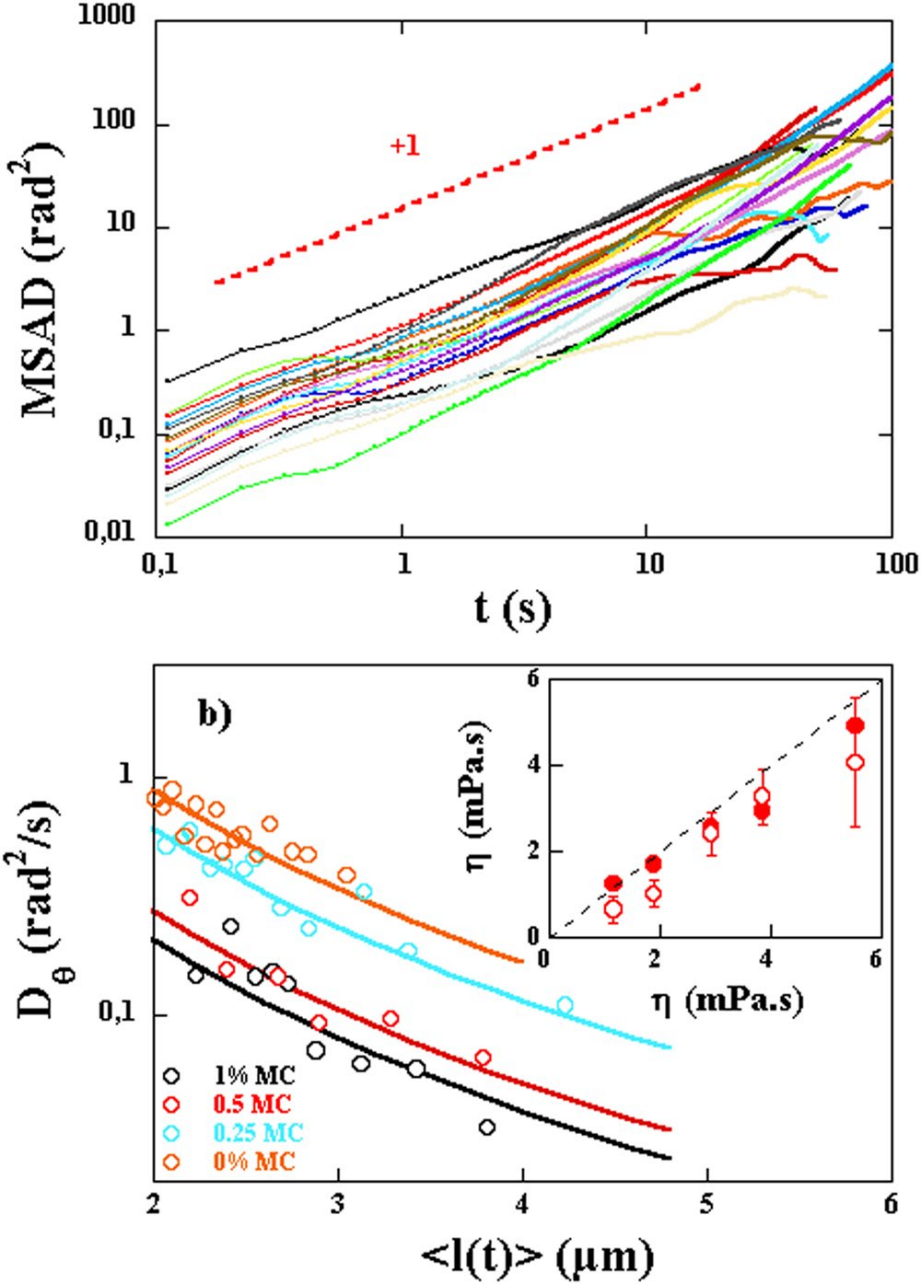
(**a**) MSAD(t) for 20 spiroplasmas in a solution of 1% MC. The dashed line shows the expected slope of 1. (**b**) The angular diffusion constant *D*_*θ*_ extracted from the MSAD(t) as a function of the mean length of the bacteria for solutions with different MC concentrations. Each measurement is an average over 10 trajectories of bacteria with similar lengths. Inset: Viscosity used to fit *D*_*θ*_ (red circles) versus the measured viscosity of the solutions used. The error bars indicate the spread in the data using different realizations of the same experiment (roughly 5). The red dots are viscosity measurements using the Brownian motion of 1.1 *μ*m diameter particles.

The simplest quantity to analyze is the angular mean square displacement. The angular mean square displacements *MSAD*(*t*) obtained by tracking individual bacteria and calculated from the temporal fluctuations of the orientation angle *θ*, are shown, for a 1% MC solution, in Fig. 5a. Each *MSAD*(*t*) is for a single bacterium. While all of them show overall linear behavior characteristic of Brownian dynamics, the value of the angular diffusion coefficient varies considerably from one bacterium to the next. This is due to the variability in the length of the tracked bacteria (eq. 8). In Fig. 5b, we show *D*_*θ*_ obtained from *MSAD*(*t*) for different lengths and different concentrations of MC. Each value of *D*_*θ*_ is obtained from 10 trajectories of bacteria with comparable lengths. The measured variation of *D*_*θ*_ is in good agreement with the expected dependence of the angular diffusion coefficient as a function of length and viscosity as given by equation 8. The fits were carried out by imposing the length and leaving the viscosity as a free parameter. The fitting value of the viscosity is then compared to the measured value using a capillary viscometer and is shown in the inset of Fig. 5b. The estimated viscosities are in good agreement with the solution viscosities indicating that the angular dynamics is given by thermal agitation and that Brownian dynamics is at play. In this inset, we also show the solution viscosity obtained using the Brownian motion of micrometer sized polystyrene beads. The obtained viscosities using this technique are also in agreement with the estimated viscosities and the solution viscosities obtained with the capillary viscometer. That the viscosities from tracking bacteria and micrometer beads as well as those from macroscopic measurements agree with each other is again another confirmation of the Newtonian aspect of the solutions used and the absence of entanglement effects^32^.

The functional form of *MSD*(*t*) is more complex. Both the characteristic velocity and the diffusion constants play a role. This functional form has two characteristic time scales: ${t}_{T}=4D/{V}_{m}^{2}$ and ${t}_{\theta }=2/{D}_{\theta }$ where $D=({D}_{//}+{D}_{\perp })/2$. The time scale *t*_*T*_ corresponds to the scale for which the contribution of motor velocity and Brownian motion are comparable while *t*_*θ*_ is the correlation time of angular fluctuations. The *MSD*(*t*) has three different regimes:

- for $t < {t}_{T}$: translational Brownian motion dominates and $MSD(t)\sim 4D\cdot t$,
- for ${t}_{T} < t < {t}_{\theta }$: the *MSD*(*t*) shows ballistic like behavior: $MSD(t)\sim {V}_{m}^{2}\cdot {t}^{2}$.
- for $t > {t}_{\theta }$: the behavior is diffusive again: $MSD(t)\sim (2\frac{{V}_{m}^{2}}{{D}_{\theta }}+4D)\cdot t$ and the effective diffusion coefficient is ${D}_{eff}={V}_{m}^{2}/2{D}_{\theta }+D$.

**Figure 6:**
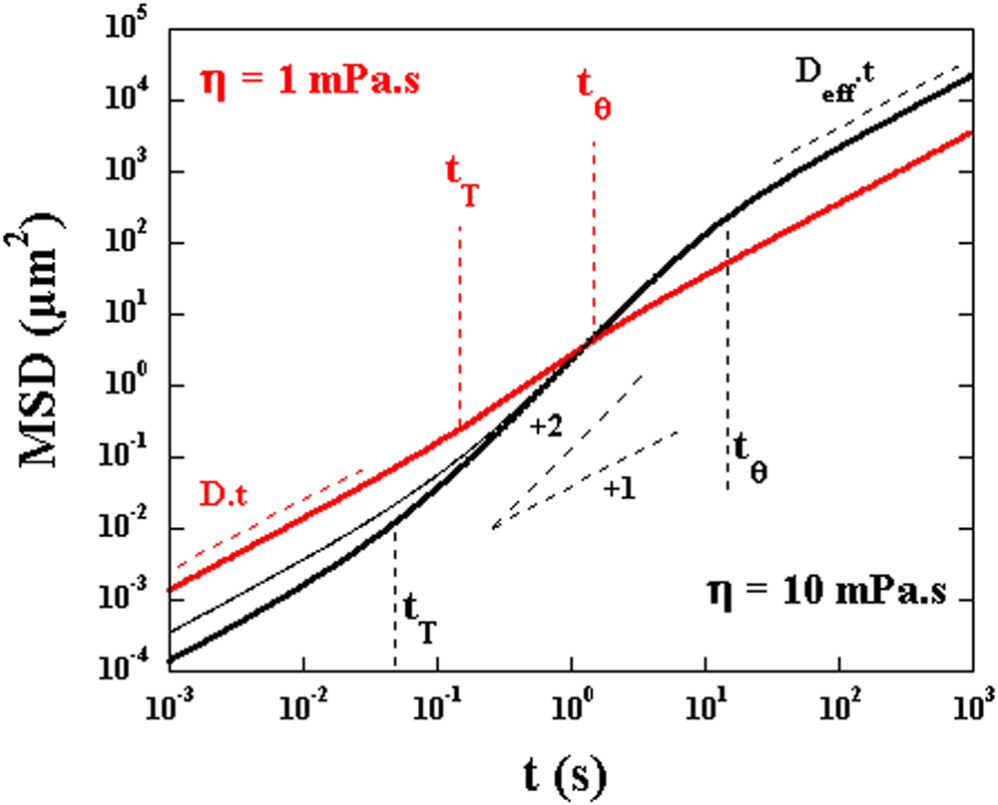
Two examples of *MSD*(*t*) for an ellipsoidal particle of length $l=3\,\mu {\rm{m}}$ and width $b=0.6\,\mu {\rm{m}}$ with a motor velocity ${V}_{m}=1.5\,\mu m/s$ for two different viscosities. The dashed lines correspond to the diffusive regime at short time scales ($t < {t}_{T}$) and the effective diffusive regime at longer time scales ($t > {t}_{\theta }$). The thin black line corresponds to the case where an additional delta correlated noise in the velocity is added. The different characteristic time scales discussed in the text are indicated along with a linear and quadratic variation of the MSD versus time.

To summarize this behavior we show a sketch of *MSD*(*t*) using equation 9 in Fig. 6 for a constant velocity ${V}_{m}=1.5\,\mu m/s$ and two different viscosities. For the large viscosity, we clearly distinguish the three regimes with ${D}_{eff}\sim 200D$ for $t > {t}_{\theta }$. But the intermediate regime, for ${t}_{T} < t < {t}_{\theta }$, becomes more difficult to distinguish at lower viscosities and the value of ${D}_{eff}\sim 3D$ is much smaller. For the small viscosity solution the transition from the first regime, for $t < {t}_{T}$, to the third regime, for $t > {t}_{\theta }$; occurs over a small interval of time (see the positions of *t*_*T*_ and *t*_*θ*_ in Fig. 6) while this interval for the higher viscosity solution extends over a longer time interval. Note here that a test with fluctuations in the velocity i.e. the presence of *u*(*t*) assumed delta correlated in time, is presented. In this case, only the diffusive part at early times, $t < {t}_{T}$, is modified.

**Figure 7:**
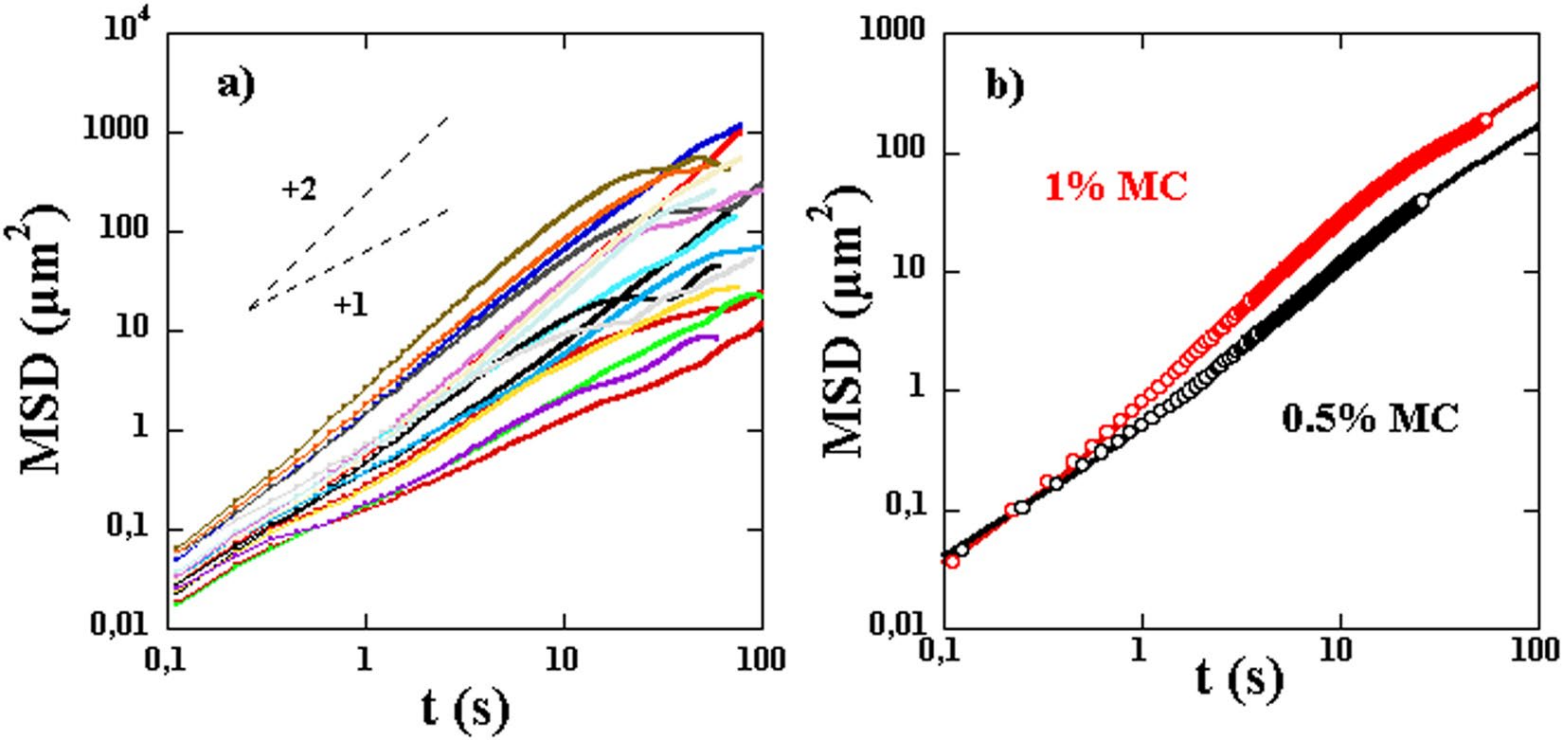
(**a**) Examples of *MSD*(*t*) for different bacteria. A linear and a quadratic regime are indicated by the dashed lines. (**b**) Average MSD(t) using 7 and 4 bacteria of roughly equal lengths for two different concentrations of MC. The fits to the expression in the text gives access to $\langle {V}_{m}(t)\rangle$. The mean velocities obtained are 0.8 and 0.4 *μm*/*s* for the high and low concentrations respectively.

**Figure 8:**
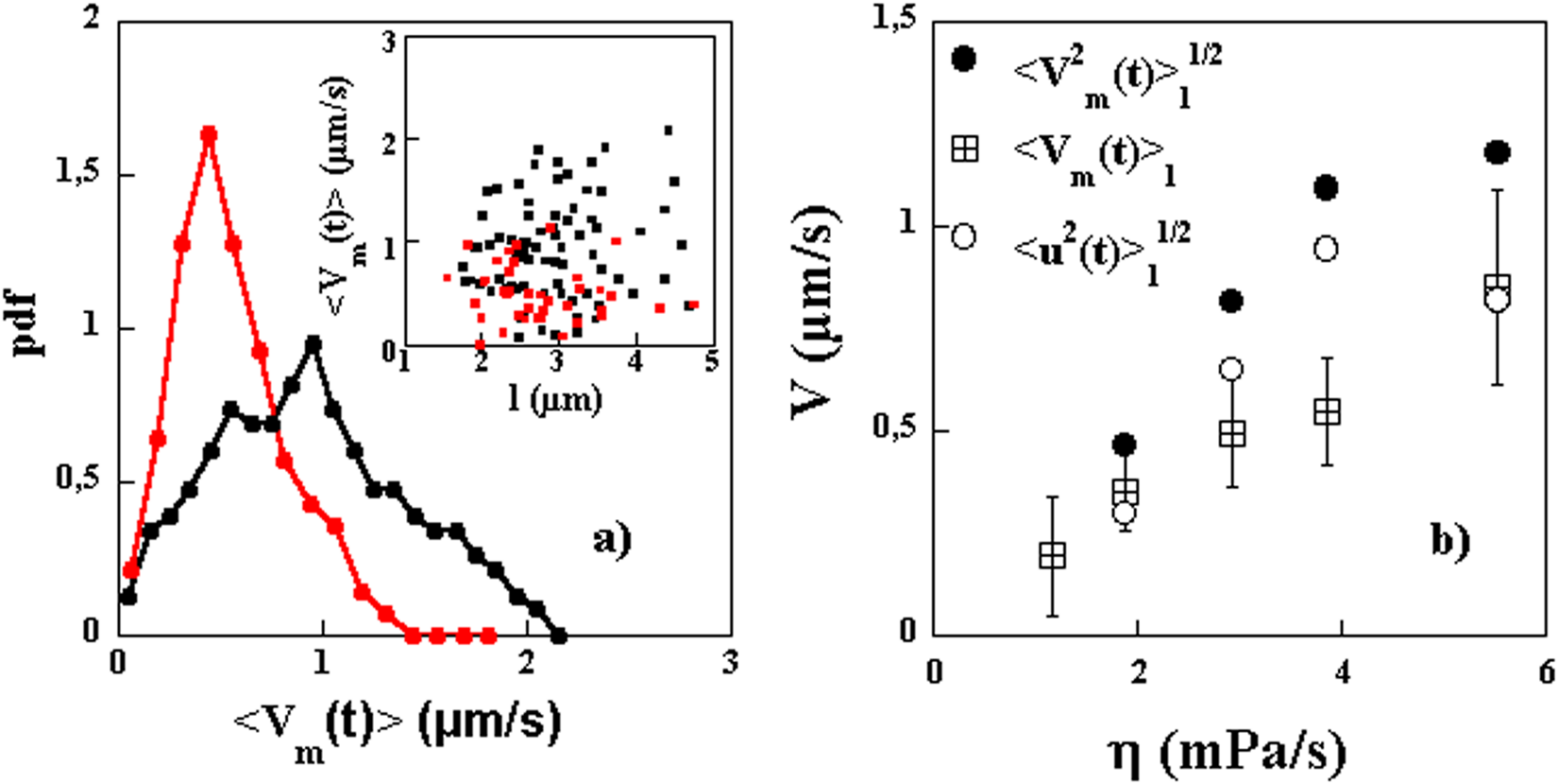
(**a**) Probability distribution functions for the mean velocity of the bacteria $\langle {V}_{m}(t)\rangle$ for two different concentrations of MC, 1% (black symbols) and 0.5% (red symbols). Inset: $\langle {V}_{m}(t)\rangle$ versus bacteria length for the two concentrations. (**b**) ${\langle {V}_{m}(t)\rangle }_{l}$ averaged over a population of bacteria of different lengths versus solution viscosity. The error bars indicate the data spread from different realizations of the same experiment (roughly 5). We have superimposed the data for ${\langle {V}_{m}^{2}(t)\rangle }_{l}^{\mathrm{1/2}}$. The difference between the two is due to the fluctuations in motor velocity ${\langle {u}^{2}(t)\rangle }_{l}^{\mathrm{1/2}}$ which is shown in the same plot.

Consider now the measured *MSD*(*t*), shown in Fig. 7a for several single bacteria. Not all of these curves show the three regimes clearly but each individual curve can be fit to the above functional form to obtain $\langle {V}_{m}(t)\rangle$ (*D*, and *D*_*θ*_ are taken as known from the above measurements). The extracted mean velocities do not seem to depend strongly on the length of the bacteria as shown in Fig. 8a where no correlation between velocity and length is observed. This is expected since these bacteria’s motility is due to the propagation of a kink and that the velocity of such kinks has not been observed to depend on the length of the bacteria^16^. However, we obtain a wide distribution of mean motor velocities showing clearly the variability from bacterium to bacterium, see Fig. 8a. These distributions depend also on the solution viscosity as shown in Fig. 8a where the distribution of $\langle {V}_{m}(t)\rangle$ is shown for two different concentrations of MC. These distributions are obtained by analyzing several trajectories from several individual bacteria. Note that the mean velocity calculated as the first moment of these distributions is higher for the higher concentration of MC and therefore a higher viscosity. This procedure works well for high viscosity solutions but is difficult to implement for low viscosity solutions where the effects of motor velocity become small.

In order to overcome this difficulty and obtain the mean velocity especially for low viscosity solutions, we show results of *MSD*(*t*) for two different concentrations of MC but averaged over several bacteria with roughly similar lengths in Fig. 7b. This averaging helps to obtain the mean velocity versus solution viscosity for low viscosity solutions more reliably. The result clearly displays the three regimes for the high viscosity solution but not for the low viscosity solution. Further, the mean values obtained, and shown in Fig. 8b, show that the mean velocity increases markedly with solution viscosity as observed previously^14–16^.

Compared to previous studies^14–16^, we find smaller motor velocities. It is possible that the difference in the values of the measured velocities is related to the way velocities are estimated. Here we measure a velocity averaged over time while in previous work, the values are measured during the passage of a kink and correspond to a phase where the bacteria have their highest velocity^16^. In fact, and from the time trace of cell positions presented in Fig. 2 of ref.^16^, we extract mean velocities in reasonable agreement with our measurements. Further, and as we showed above, the motor velocity of the bacteria may show large velocity fluctuations in time. The measurements shown in Fig. 8b are for the mean velocity excluding these fluctuations. In the method used above, both the intensity of these fluctuations and the mean velocity were a priori measured in the form of ${\langle {V}_{m}^{2}(t)\rangle }^{1/2}$. Figure 8b shows a comparison between the mean velocities $\langle {V}_{m}(t)\rangle$ and the characteristic velocities ${\langle {V}_{m}^{2}(t)\rangle }^{1/2}$, these two quantities differ by almost a factor of two. The difference $\langle {V}_{m}^{2}(t)\rangle -{\langle {V}_{m}(t)\rangle }^{2}=\langle {u}^{2}(t)\rangle$ can be used to estimate the amplitude of the fluctuations which turns out to be as important as $\langle {V}_{m}(t)\rangle$ if not more. The amplitude of these fluctuations is shown in Fig. 8b.

In the preceding sections, we have therefore shown that the mobility of the bacteria is controlled by their motor velocity and by Brownian motion. This motor velocity has large variability from one bacterium to the next and large temporal variability. While the analysis of the bacteria’s mean square displacement gave us access to the mean velocity of the bacteria, the angular analysis of the velocity gave us access to the temporal variability. The main result is that this temporal variability is strong and both the mean velocity and the amplitude of the fluctuations increase as the viscosity increases. In the following, we will focus on the movement of a single bacterium and analyze its temporal dynamics by focusing both on the variations in its orientation and its instantaneous velocity. By using long trajectories where both the orientation and the position of a single bacterium can be tracked for long periods of time without the bacterium leaving the focal plane and without reorienting out of the plane of the measurement, we will illustrate the origin of these temporal fluctuations.

## Properties of the Trajectories of Individual Bacteria

**Figure 9:**
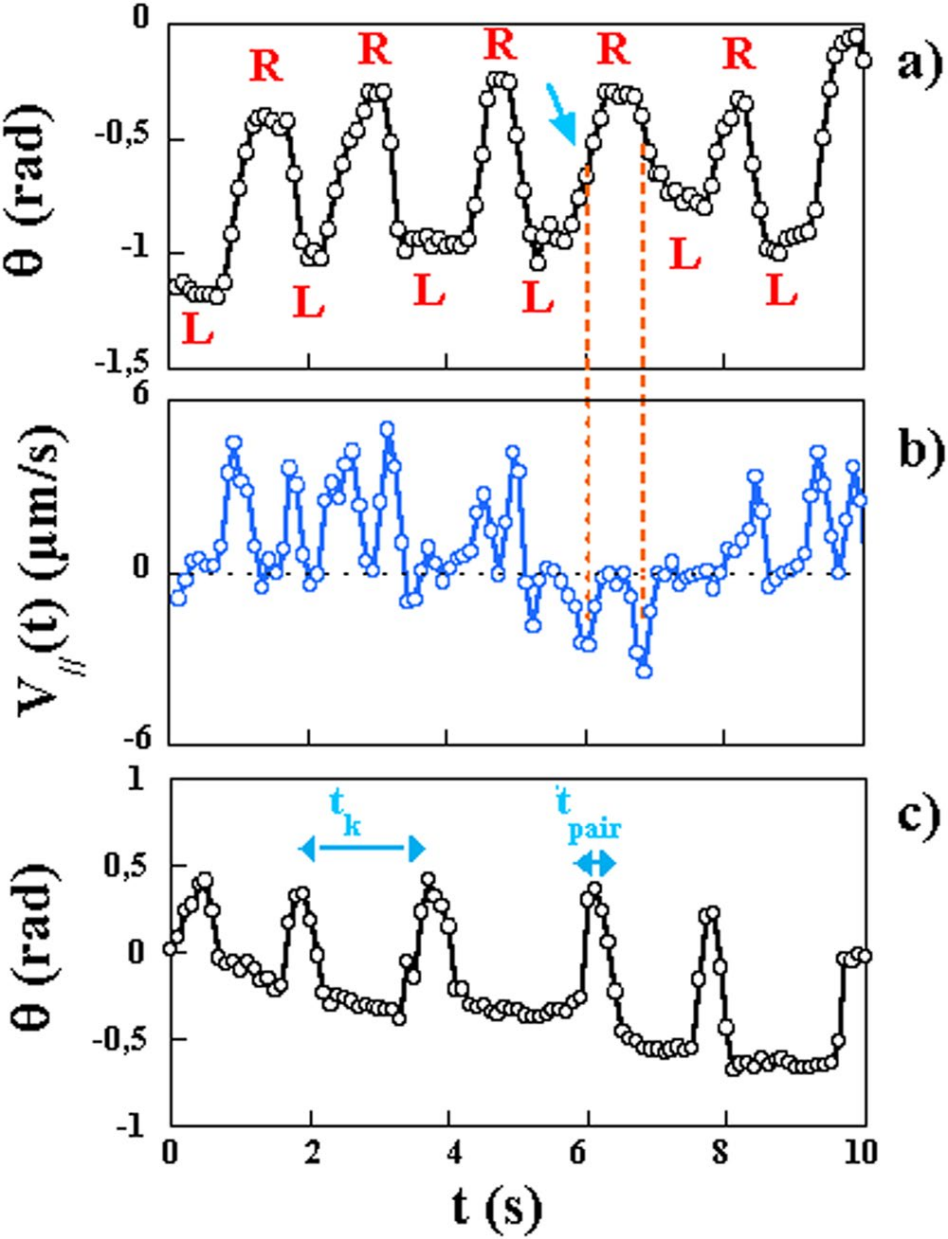
(**a**) Plot of *θ*(*t*) for a single bacterium in a solution of 1% MC. The letters *R* (right) and *L* (left) indicate the helicity of the bacterium at each plateau value of the orientation. (**b**) Variation of the velocity of the bacterium in the direction of the long axis ${V}_{//}(t)$ for the same bacterium. Note that on rare occasions the bacterium moves backwards: The blue arrow indicates this start event and the orange dashed lines show that the maximum velocity is negative. (**c**) Another example of the variation of the orientation *θ*(*t*). The time *t*_*pair*_ is defined as the time between the passage of two successive kinks while *t*_*k*_ is the time interval between the passage of two pairs of kinks.

In order to further study the variability in mobility, we here analyze, in detail, the trajectories of individual bacteria in the more viscous case (1% MC). We only analyze trajectories for which the bacteria remain in the plane of observation so that the two ends of the bacteria can be followed in time continuously. This limits the analysis to fewer trajectories and to viscous solutions only compared to the statistical analysis carried out above. Nevertheless, such analysis of individual trajectories and for long periods of time allows to get more insight into the mobility of these bacteria. We have paid particular attention to the presence and displacement of kinks along the bacteria as has been described in^14,16^. The analysis carried out below is for short bacteria with lengths *l* < 4. *μ*m for which we rarely observe the propagation of two kinks simultaneously and which renders the analysis simpler. The analysis we carry out in this section on single bacteria relies mainly on tracking the individual bacterium to measure its instantaneous center of mass velocity $\overrightarrow{V}$ and its main orientation *θ*. We have visually inspected the images to identify the two ends of the bacterium and determine its orientation. We also measure the helicity of the bacterium especially during the propagation of a kink. Examples of these measurements are shown for *θ* and ${V}_{//}=\overrightarrow{V}\cdot \overrightarrow{n}$ (in the direction $\overrightarrow{n}$) in Fig. 9a,b. The orientation *θ* of the bacterium oscillates between two plateau values with a typical period of roughly 1 *s*. The plateau values differ by roughly 45° and are followed by short periods of rapid variation of the orientation. These short periods last roughly 0.2 to 0.3 *s*. The succession of plateaus in the orientation of the bacterium is directly correlated to a change in helicity of the bacterium.

**Figure 10:**
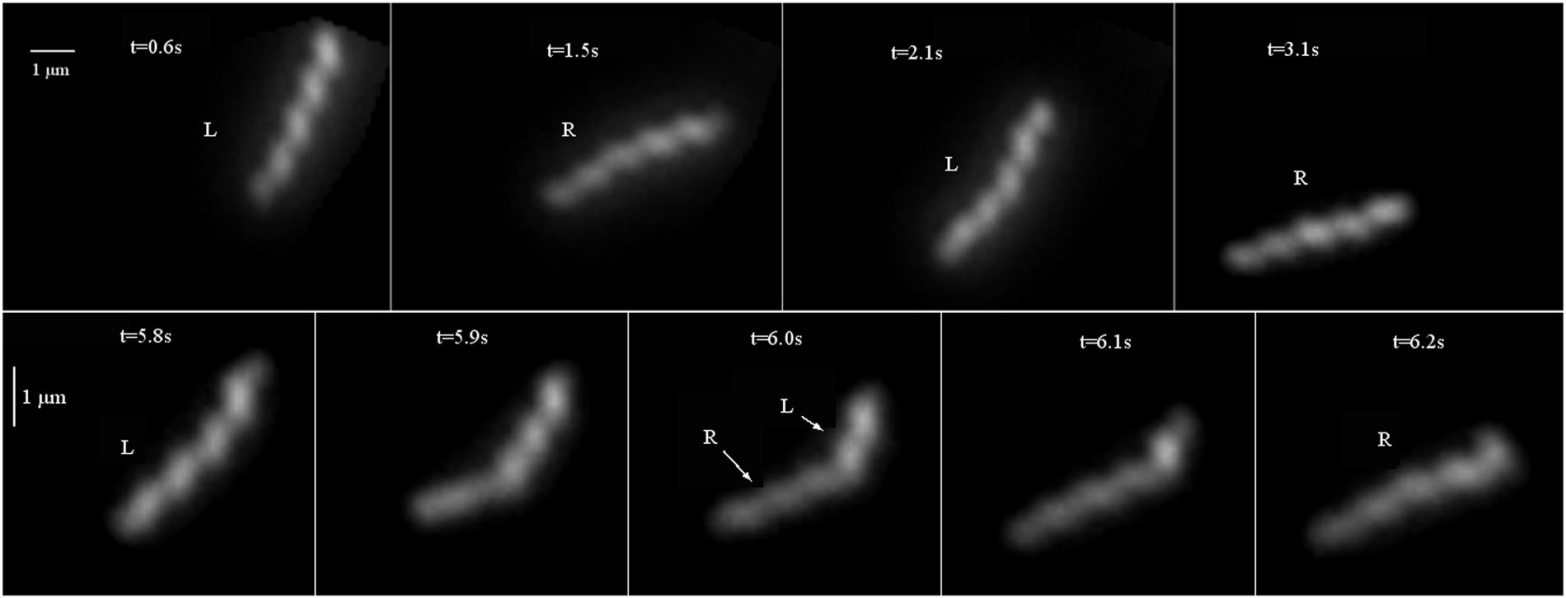
Upper sequence: images of a bacterium. Note the change in helicity and orientation between two images. Bottom sequence: the passage of a kink is accompanied by a change of helicity (L, left, R, right) and orientation of the bacterium.

The sequence of images in Fig. 10, which correspond to the time trace shown in Fig. 9a,b, shows the bacterium at different instants corresponding to the two plateaus of orientation. The variation of intensity along the bacterium gives a hint as to its helicity. The bacterium at t = 0.6 s is left handed while that at t = 1.5 s (which is roughly 1 s later and is in the lower plateau) is right handed. The two plateaus in orientation correspond to the two helicity states of the bacterium. The bottom sequence of images, Fig. 10, shows the short time dynamics of the change in orientation from one plateau value to the next. During this short period of time, a kink propagates along the bacterium and a change of helicity occurs during this period giving the alternate helicity for the lower plateau in orientation. The propagation of a kink is responsible for the rapid change in orientation from one plateau value to the other and is accompanied by a change of the helicity of the bacterium as has been suggested previously^14,16^. Note that for these images and for the analysis of Fig. 9, care was taken to ensure that the observations are carried out from above the helix axis.

Let us come back to the velocity time trace shown in Fig. 9b. This time trace is directly correlated to the angular variations *θ*(*t*) The velocity is highest (roughly 5 *μm*/*s*) during the rapid variations in orientation while in the plateaus, the velocity is very small. This observation confirms that the bacterium moves during the phase of kink propagation with the bacterium moving in the opposite direction to that of the kink. While most events propelled the bacterium in the same direction, a few events push the bacterium backwards as seen in Fig. 9b where a couple of large negative velocities were observed.

Previous work^16^, found that the velocity of propagation of a kink is roughly constant ${v}_{kink}=10.3\,\mu m/s$ independent of the viscosity of the medium. Also, kinks come in pairs separated by a time *t*_*pair*_. This time scale has a mean value of 0.26 *s*. The time between two such pairs *t*_*k*_ was found to have a wide distribution which decays approximately exponentially with a time constant of the order of a second^16^. Our own observations confirm these previous measurements. We find ${v}_{kink}=12\pm 2\,\mu m/s$. The time scale *t*_*k*_ has variability both in time and from bacterium to bacterium. Another example of the variation of the orientation is shown in Fig. 9c. Note that in this example the values of the two time scales are different from the example shown above and that these time scales vary with time.

**Figure 11:**
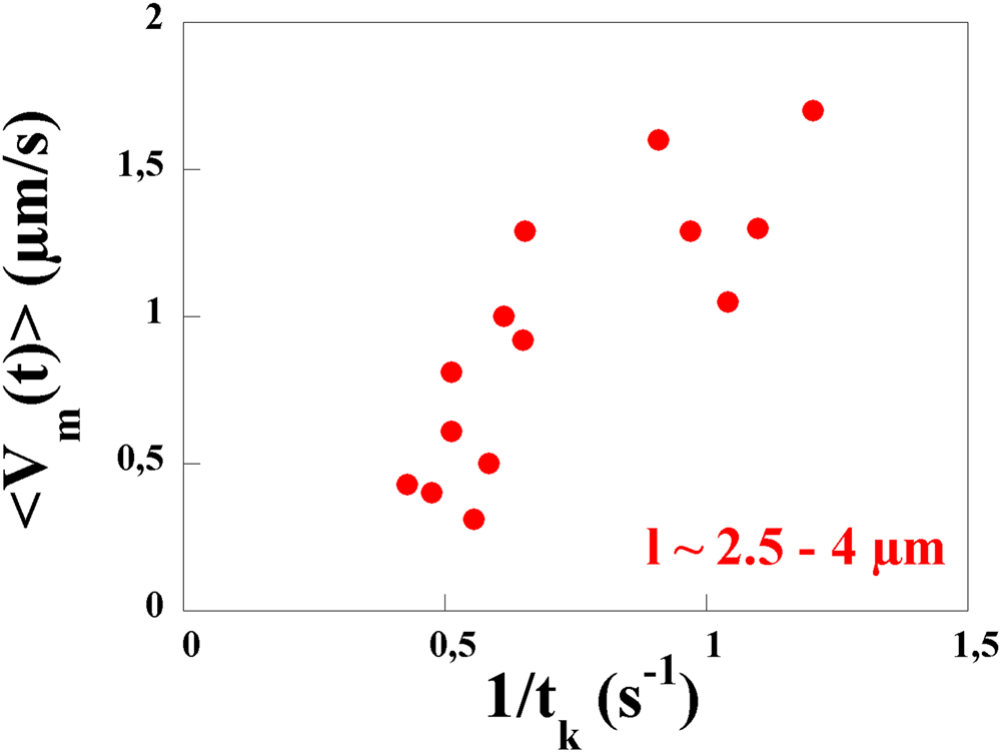
The velocity $\langle {V}_{m}(t)\rangle$ versus 1/*t*_*k*_ in a solution of 1% MC. The measurements are carried out using only sequences of images where the kinks travel in the same direction.

It has already been noted that the passage of the kink is responsible for the motility of the bacteria^14,16,33–35^. In addition, our results show that the instantaneous velocity of the bacteria has large fluctuations with short periods of fast mobility and periods of slow movement. The time scale *t*_*k*_ and the mean velocity of the bacteria are related in a simple way. Suppose that as a pair of kinks propagates along the bacterium, the cell moves a distance 2*λ* (one *λ* for each kink and assuming that the two kinks have moved in the same direction). The mean velocity $\langle {V}_{m}(t)\rangle$ obtained from time traces such as those of Fig. 9 can then be approximated as 2*λ*/*t*_*k*_. Figure 11 shows a plot of this mean velocity $\langle {V}_{m}(t)\rangle$, obtained from 15 different tracks, versus 1/*t*_*k*_ for a solution of 1% MC. The measurements show a roughly linear correlation between the two quantities with a slope of 1.4 giving *λ* ~ 0.7 *μm*. This type of measurement was more difficult to carry out for lower viscosity solutions.

**Figure 12:**
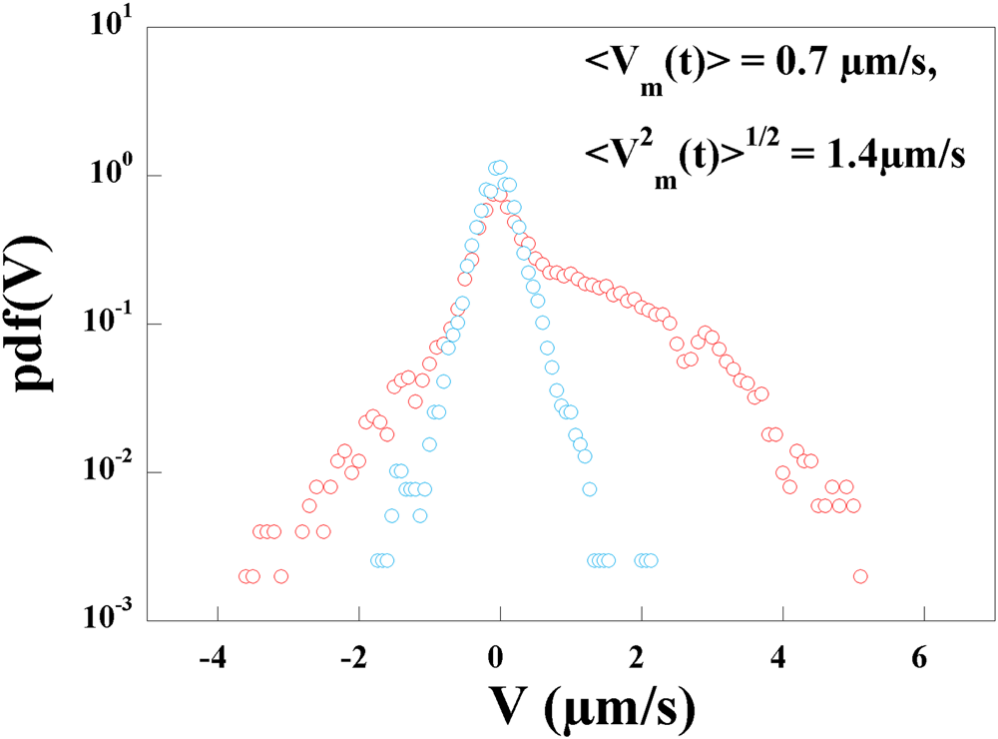
Probability Density Functions of ${V}_{//}(t)$ (red circles) and ${V}_{\perp }(t)$ (blue circles) in a solution of 1% MC.

Figure 11 was obtained from image sequences of trajectories where the kinks emanated from the same bacterium end assuring that the mobility was always in the same direction. This is not always the case as kinks in the opposite direction can also be observed as seen in Fig. 9. In order to appreciate this effect, we plot in Fig. 12 the probability density functions of ${V}_{//}(t)$ (red circles) and ${V}_{\perp }(t)$ (blue circles, in the direction perpendicular to the main axis) for a bacterium which was followed for 160 *s* at a frame rate of 10 images per second. The distribution of ${V}_{\perp }(t)$ is as expected: it is symmetric and centered around 0. The width of this distribution is solely due to Brownian motion. The distribution of ${V}_{//}(t)$ has different contributions coming from Brownian translational motion as well as the motor velocity *V*_*m*_(*t*). This distribution is more complex with a central peak due to Brownian motion and roughly similar to that of the transverse velocity. This distribution is however asymmetric with positive velocities that are more probable than negative velocities and is very broad. While positive velocities are associated with kinks moving forward, on a few occasions kinks may propagate in the opposite direction giving negative velocities but these events are not as frequent.

## Discussion and Conclusion

By tracking individual spiroplasmas we have shown that they move in the direction of their long axis with a motor velocity which presents a large variability from one bacterium to the next and a large temporal variability. From a statistical analysis using a Langevin equation for both the translational and the rotational dynamics of the bacteria approximated as ellipsoids, the rotational motion was found to follow Brownian dynamics with angular diffusion coefficients determined by the size of the bacteria and the viscosity of the medium. This statistical analysis allowed us to disentangle the Brownian motion from the motor velocity and its fluctuations. This motor velocity was found to increase with solution viscosity. The amplitude of the temporal fluctuations of the motor velocity were also obtained and found to be large and comparable to the mean motor velocity. This temporal variability is due to the way these bacteria are propelled. This propulsion is controlled by the passage of kinks that travel from one end of the bacterium to the other. The passage of each kink gives rise to a large variation of the orientation of the bacterium and propels it forward in a short time period. Once the kink has propagated from one end to the other, the bacterium remains roughly static. It is this process of mobility, large movements followed by periods of rest which give rise to large temporal variations in the velocity of the bacteria. The kinks actually come in pairs, which propagate in the same direction and are separated by a short time interval. The time period between the passage of pairs of kinks is longer and is correlated to the mean velocity of these bacteria. An interesting point here is the presence of long waiting times between the passage of pairs of kinks. These times and their distribution is probably an intrinsic property of the motility of these bacteria. This waiting time maybe simply related to the time necessary for recharging the intracellular ATP pool and allow the bacteria to find the necessary energy for a subsequent kink generation. These waiting periods or pausing could enable the cell reorientation by Brownian motion for example. The variability from one bacterium to the next is probably a consequence of the variability in the time period between the passage of pairs of kinks; it is possible that the mean period is specific to each bacterium.

The proposed analysis here allows to disentangle the different contributions to the mobility of these bacteria: Brownian motion and motor velocity. The analysis also allows to unambiguously show the variability in motor velocity from bacterium to bacterium. The mean velocity as well as the amplitude of the temporal fluctuations of the velocity both increase with the medium viscosity. In fact, for low viscosity fluids (near the viscosity of water), Brownian motion is dominant and the motor velocity is small. Using such a quantitative approach may help in characterizing the motility of variants of *S*. *citri* for example to explore links between their mobility and their pathogenicity.

One of the main questions raised by our analysis concerns the mechanisms behind the enhancement of the swimming speed versus viscosity. It has been argued before that such an enhancement is due to viscoelastic effects and possible entanglement of the polymer solutions^32,33,36^. The polymer solutions used here are however Newtonian considering the small molecular weight of the polymer and the concentrations used (all in the dilute regime). Different diagnostics point to this fact including the shear rheology and the microrheology used here. In fact, and in order for solutions of MC to acquire Non Newtonian behavior, high molecular weights are required^37^. Consider for example the determination of the viscosity using Brownian particles of roughly 1 micron in diameter. Their mean square displacement was found to be given by the viscosity of the solution measured using a capillary viscometer or a rheometer. Further, the diffusion constants of the bacteria (whether for rotational diffusion or translational diffusion) are given by the viscosity of the solution. Basically, microrheology measurements show that no anisotropy is observed and that the solution properties are governed by the viscosity of the solution as measured on a macroscopic scale^32^. This, coupled to the linearity of the viscosity versus concentration of the solutions used, all indicate that Non-Newtonian effects as well as entanglement effects can be excluded. Few mechanisms have been proposed to explain this enhanced swimming speed without invoking the entangled nature of the solution or its non Newtonian character. Perhaps the most relevant is depletion effects of polymers near the bacterium which coupled to the rotational motion of the bacterium may give swimming speed enhancement as proposed in^38^. However, this remains to be tested carefully.
